# A metastatic ureteral tumor successfully treated with multidisciplinary therapy including radiotherapy

**DOI:** 10.1002/iju5.12582

**Published:** 2023-03-03

**Authors:** Yoshihiro Kawaguchi, Kazuya Naritomi, Nobutoshi Kawagoe, Yoshihiro Matsunaga, Naoyuki Ogasawara, Tsukasa Igawa

**Affiliations:** ^1^ Department of Urology Saiseikai Futsukaichi Hospital Chikushino‐shi Fukuoka Japan; ^2^ Department of Surgery Saiseikai Futsukaichi Hospital Chikushino‐shi Fukuoka Japan; ^3^ Nakamura Clinic Chikugo‐shi Fukuoka Japan; ^4^ Department of Urology Kurume University School of Medicine Kurume Fukuoka Japan

**Keywords:** gastric cancer, radiotherapy, signet ring cell carcinoma, ureteral metastasis, ureteroscopy

## Abstract

**Introduction:**

Metastatic ureteral tumors are difficult to diagnose pathologically. Treatment is only available for the primary disease, and prognosis is generally poor.

**Case presentation:**

A 63‐year‐old patient with a history of gastric cancer presented with asymptomatic right‐sided hydronephrosis. Ureteroscopic examination revealed tissue in the ureter consistent with gastric cancer. The lesion was localized, and the patient was treated with chemotherapy and radiotherapy as part of a multidisciplinary treatment. The prognosis was better than in other reports. To the best of our knowledge, this is the first case of a patient with metastatic gastric cancer who received multidisciplinary treatment including radiotherapy and had a good prognosis.

**Conclusion:**

In cases where a localized metastatic ureteral tumor cannot be ruled out, ureteroscopy is an effective therapeutic strategy.

**Abbreviations & Acronyms**
PET‐CT: positron emission tomography‐computed tomography
PTX: paclitaxel
RAM: ramucirumab
SP: S‐1 and cisplatine
TURBT: transurethral resection of bladder tumor

**Keynote message**

Metastatic ureteral tumors are difficult to diagnose and have few treatment options. There are few reports of successful treatment. When a localized metastatic ureteral tumor cannot be ruled out, more aggressive ureteroscopy may be a useful therapeutic strategy.

## Introduction

Metastatic ureteral tumors are difficult to diagnose pathologically. The only treatment is according to the primary disease, and the prognosis is generally poor.^1^ We report a case in which metastatic gastric cancer was diagnosed by ureteroscopy and treated with radiotherapy as part of a multidisciplinary treatment program with a relatively good prognosis. Ureteroscopy is useful for diagnosis and selecting a treatment strategy.

## Case presentation

**Fig. 1:**
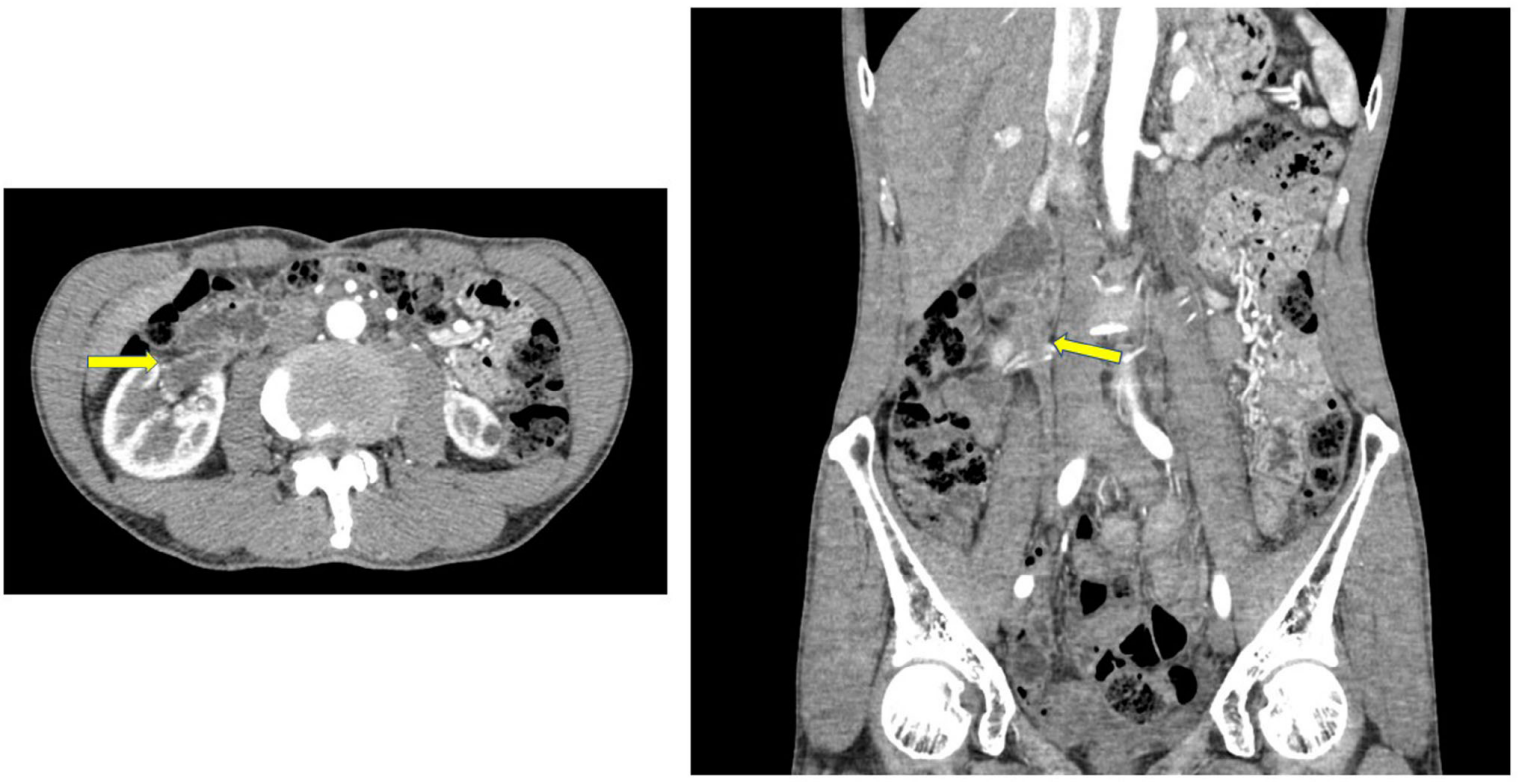
Contrast‐enhanced computed tomography scan showing dilatation, wall thickening, and irregularity of the right renal pelvis and upper ureter (arrow).

**Fig. 2:**
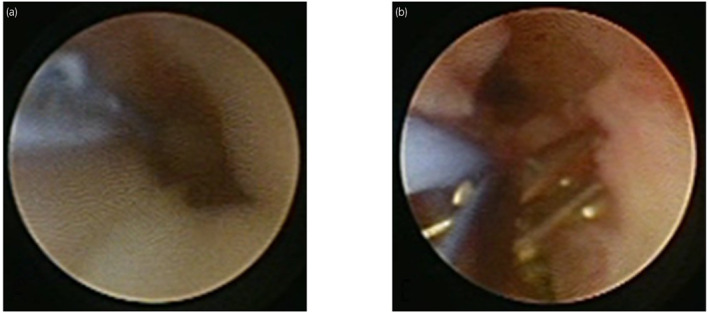
(a) Circumferential stenosis was observed in the upper ureter, and insertion of a rigid ureteroscope upstream of the stenosis was not possible. (b) Biopsy was performed from the same site with biopsy forceps.

**Fig. 3:**
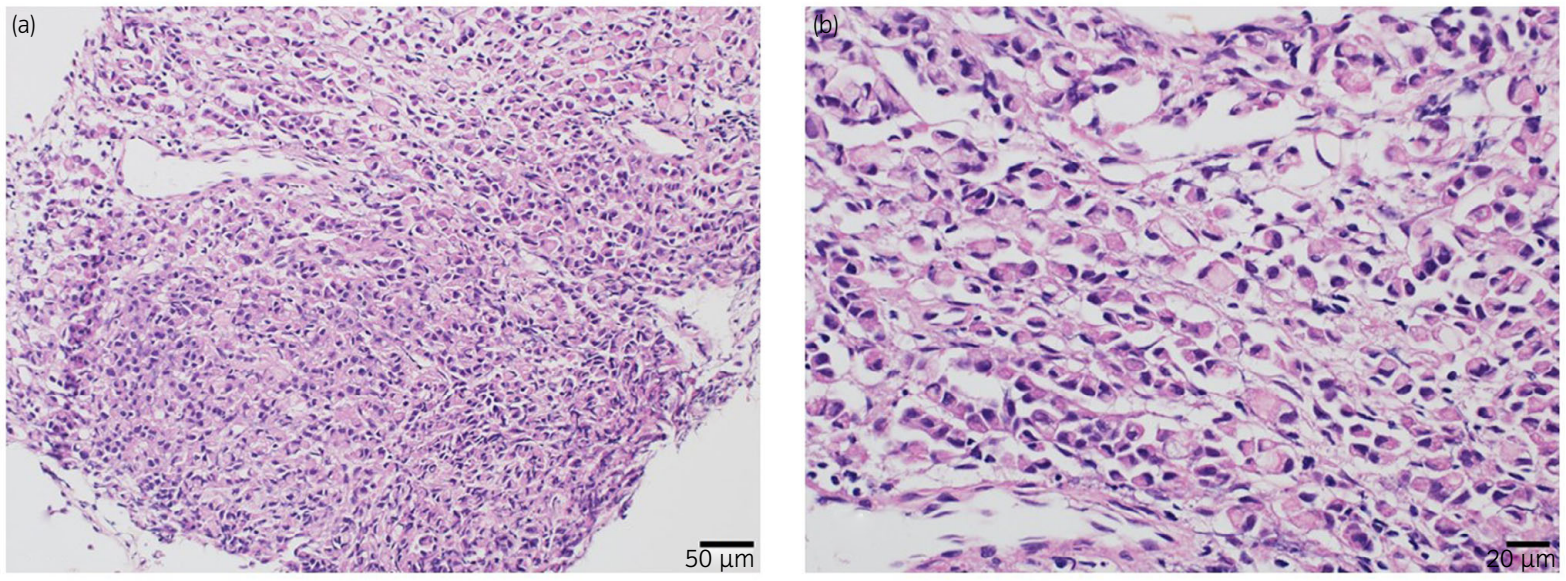
Histopathological findings with (a) median (magnification 100×) and (b) high‐power (magnification 400×) images after hematoxylin and eosin staining showing numerous indolent annular cell carcinomas with unevenly distributed nuclei and mucus retention, similar to those in the gastric biopsy tissue.

**Fig. 4:**
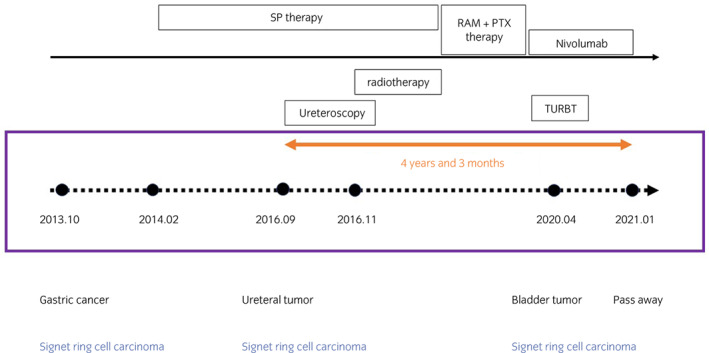
Chart diagram with timeline of therapy and treatment. The prognosis is shown 4 years and 3 months after the diagnosis of metastatic ureteral tumor.

In 2016, a 63‐year‐old male presented with right hydronephrosis and was referred to the Urology Department (Fig. 1), with absent right lumbar back pain or gross hematuria. In 2009, he underwent gastric cancer surgery (pT1bN0M0), pathologically identified as moderately differentiated adenocarcinoma. In 2013, he underwent further surgery for residual gastric cancer recurrence (pT4aN1(1/6) stage IIIA por/sig). SP therapy was initiated as anticancer treatment for advanced carcinoma with heterochronic gastric cancer. Urological examination revealed abnormal blood test results, including mildly elevated carcinoembryonic antigen (8.7 ng/mL; normal range 0–5.0 ng/mL) and mildly lowered creatin (1.12 mg/dL; normal range 0.65–1.07 mg/dL) levels. However, a PET‐CT scan showed no abnormalities, and class I urine cytology. As the possibility of a primary ureteral tumor could not be excluded, ureteroscopy with ureteral stenting was performed. The upper urinary tract had a circumferential stenosis, which prevented insertion of the rigid ureteroscope (Fig. 2). Isolated urine cytology was identified as class II, and pathological examination identified signet ring cells (Fig. 3). Given preoperative evidence of renal pelvis dilation, ureteral stenting was also performed. PET‐CT was repeated; however, no abnormalities were detected. With no other options available, the lesion was localized and high‐grade cancer was detected, thus radiation therapy was added as a quasi‐curative treatment. External radiation extending from the right kidney to the pelvic ureter was initiated as part of a multidisciplinary treatment (radiation was administered 28 times; total radiation dosage of 50.4 Gy), after which SP therapy was continued. After 13 courses of SP therapy, the regimen was changed to RAM and paclitaxel (RAM + PTX) due to side effects. Routine CT scrutiny revealed no new lesions. In 2020, the patient presented with asymptomatic gross hematuria and underwent TURBT (c), which revealed signet ring cell carcinoma. After TURBT, CT scans revealed right ureteral and periaortic lymph nodes and liver and bone metastases. The patient received a total of seven courses of RAM+PTX for advanced gastric cancer and treatment with nivolumab; however, he died in January 2021, 4 years and 3 months after ureteral metastasis diagnosis (Fig. 4).

## Discussion

This case study sheds light on two important considerations for clinical care. First, ureteroscopy could be used for ureteral tumor diagnosis from other organs; second, diagnosis by ureteroscopy may lead to appropriate treatment, including multidisciplinary treatment. The treatment strategies for primary and metastatic ureteral tumors differ greatly, and gastric cancer diagnosis allowed selection of an appropriate treatment plan. After metastatic ureteral tumor diagnosis, multidisciplinary treatment, including radiotherapy, resulted in a prognosis of 4 years and 3 months. Periodic stent replacement was performed due to the presence of a dilated renal pelvis and the risk of worsening ureteral stricture after radiation, as reported in other cases.^2^

Metastatic ureteral tumors are very rare and were first reported in 1909 by B. Stow.^3^ On imaging, it is difficult to distinguish primary from metastatic ureteral tumors.^4^ Gastric cancer can metastasize by lymphatic, hematogenous, or peritoneal dissemination. The exact form of metastasis is clinically unknown in this case without surgical resection or autopsy. PET‐CT before and after ureteroscopy showed no abnormal findings. There was no direct invasion from the primary tumor, and the same tumor cells were found in the primary tumor and part of the ureteral wall, meeting the definition of metastatic ureteral tumor by Pressman and Murayama et al.^5^ Chemotherapy is recommended as salvage therapy for advanced gastric cancer, but radiation therapy is rarely mentioned. Radiotherapy has no evidence, and it is impossible to determine whether radiation, chemotherapy, or multidisciplinary treatment was more effective in this case. Approximately 90% of metastatic ureteral tumors metastasize to other organs, and approximately 75% of patients die within 6 months.^6^ Advanced or metastatic gastric cancer has a 5‐year survival rate of 5%–20%, with a median overall survival of <1 year.^1^ Immune checkpoint inhibitors as a new treatment option have shown overall prolonged survival but have not yet achieved long‐term prognosis.^7^ In this case, where a localized metastatic ureteral tumor was undeniable, the diagnosis was made by ureteroscopy, leading to appropriate treatment.

## Author contributions

Yoshihiro Kawaguchi: Conceptualization; data curation; formal analysis; methodology; project administration; resources; visualization; writing – original draft; writing – review and editing. Kazuya Naritomi: Project administration; writing – review and editing. Nobutoshi Kawagoe: Project administration; writing – review and editing. Yoshihiro Matsunaga: Project administration; writing – review and editing. Naoyuki Ogasawara: Project administration; writing – review and editing. Tsukasa Igawa Project administration; writing – review and editing.

## Conflict of interest

The authors declare no conflict of interest.

## Informed consent

Written informed consent was obtained from the patient for publication of this case report.

## Registry and the registration No. of the study/trial

Not applicable.

## Approval of the research protocol by an institutional review board

Not applicable.

## Funding information

This case did not receive any specific grants from funding agencies in the public, commercial, or not‐for‐profit sectors.

## Data Availability

The data is available from the corresponding author upon reasonable request.

## References

[1] Bang YJ , Van Cutsem E , Feyereislova A *et al*. Trastuzumab in combination with chemotherapy versus chemotherapy alone for treatment of HER2‐positive advanced gastric or gastro‐oesophageal junction cancer (ToGA): a phase 3, open‐label, randomized controlled trial. Lancet 2010; 376: 687–97. 20728210 10.1016/S0140-6736(10)61121-X

[2] Straub JM , New J , Hamilton CD , Lominska C , Shnayder Y , Thomas SM . Radiation‐induced fibrosis: mechanisms and implications for therapy. J. Cancer Res. Clin. Oncol. 2015; 141: 1985–94. 25910988 10.1007/s00432-015-1974-6 PMC4573901

[3] Stow B . Fibrolymphosarcomata of both ureters metastatic to a primary lymphosarcomata of the anterior mediastinum of thymus origin. Ann. Surg. 1909; 50: 901–6. 17862436 10.1097/00000658-190911000-00009 PMC1407201

[4] Karaosmanoglu AD , Onur MR , Karcaaltincaba M , Akata D , Ozmen MN . Secondary tumors of the urinary system: an imaging conundrum. Korean J. Radiol. 2018; 19: 742–51. 29962880 10.3348/kjr.2018.19.4.742 PMC6005933

[5] Ebine T , Shindo M , Fujita K , Mikami S . Metastatic ureteral tumor from colon cancer: a case report. Hinyokika Kiyo 2014; 60: 143–6. 24759502

[6] Arvind NK , Singh O , Gupta S , Ali Q . Ureteral metastasis as the presenting manifestation of pancreatic carcinoma. Rev. Urol. 2013; 15: 124–30. 24223025 PMC3821992

[7] Kang YK , Boku N , Satoh T *et al*. Nivolumab in patients with advanced gastric or gastro‐oesophageal junction cancer refractory to, or intolerant of, at least two previous chemotherapy regimens (ONO‐4538‐12, ATTRACTION‐2): a randomized, double‐blind, placebo‐controlled, phase 3 trial. Lancet 2017; 390: 2461–71. 28993052 10.1016/S0140-6736(17)31827-5

